# ID 334 - Teleconsultoria como Tecnologia para Equidade na Acessibilidade ao Cuidado em Saúde com Qualidade

**DOI:** 10.5327/2237-9622.2025.v34s1.334

**Published:** 2025-11-25

**Authors:** Maria Aparecida de Assis Patroclo, Higor João de Miranda

**Keywords:** teleconsultoria, revisão de escopo, objetivos, desenhos de estudos, desfechos curricularização da extensão

## Abstract

**Introdução:**

A telemedicina é definida como "o exercício da Medicina através da utilização de metodologias interativas de comunicação audiovisual e de dados, com o objetivo de assistência, educação e pesquisa em Saúde", englobando a TELECONSULTORIA, que é a interação entre profissionais da saúde para fins de assistência ou educacional de forma síncrona ou assíncrona (MS, 2020). Em 2002, o Conselho Federal de Medicina regulamentou a prática de telemedicina no Brasil, sendo introduzida no Sistema Único de Saúde (SUS) em 2007. Este estudo pretende convocar os Núcleos de Avaliação de Tecnologias em Saúde (Nats) dos hospitais universitários a reflexão que, para além de avaliações considerando o alto custo e o risco, é importante que nesse tipo de instituição a avaliação e o monitoramento do horizonte tecnológico contribuam para que o ensino, a extensão e a pesquisa cumpram seu papel social. O principal objetivo foi realizar uma revisão de escopo sobre teleconsultoria como tecnologia para equidade na acessibilidade ao cuidado em saúde com qualidade.

**Método:**

Revisão de escopo é definida como um tipo de síntese de evidências para identificar e mapear, de forma sistemática e ampla, as evidências disponíveis sobre um determinado tema. Definiu-se como população os profissionais de saúde, como conteúdo a teleconsultoria como tecnologia para melhoria do cuidado em saúde e, em relação ao contexto, não houve critério de exclusão. Os estudos foram descritos, sintetizados os principais aspectos, feitas a discussão e as recomendações, seguindo as diretrizes do Joanna Briggs Institute.

**Resultados:**

Foram identificadas 1.645 referências, das quais 32 foram selecionadas para análise e distribuídas em 4 grupos: teleconsultoria para médicos; teleconsultoria para médicos e outros profissionais não médicos; teleconsultoria para outros profissionais de saúde; e teleconsultoria para exames e outras solicitações de profissionais. A teleconsultoria tem sido adotado em países de baixa, média e alta renda, tendo os estudos analisados sido desenvolvidos no Nepal, no Brasil, na Alemanha, no Canadá, na Escócia, nos Estados Unidos, na França, na Itália, na Noruega e nos Países Baixos. Os objetivos mais prevalentes foram avaliar a aceitabilidade, os custos, a eficácia, a efetividade, a eficiência, a segurança e a viabilidade da teleconsultoria, os benefícios, os impactos e o sucesso da implementação, a satisfação de médicos e pacientes, entre outros. Os desenhos de estudo mais frequentes foram estudos experimentais, observacionais e relato de experiências.

**Conclusão:**

As evidências encontradas demonstraram o alcance de desfechos favoráveis em relação à oferta de cuidado com qualidade; ao uso e ao potencial da tecnologia; à contribuição para a educação médica e de outros profissionais; à melhoria da comunicação com especialistas e na equipe; à redução de tempo de espera e de custos; e à satisfação dos profissionais e pacientes. A revisão de escopo permitiu um mapeamento de estudos avaliativos sobre teleconsultoria, revelando as principais diretrizes para implantação e implementação da tecnologia para alcance de sucesso em diferentes contextos. A adoção do uso de teleconsultoria em projetos com vistas à curricularização da extensão em faculdades da área da saúde e hospitais universitários tem a capacidade de integrar docentes, estudantes e técnicos aos diferentes níveis de atenção e o potencial de produzir impacto social ao reduzir a morbimortalidade ao permitir a maior equidade na acessibilidade a atendimento de qualidade com apoio de especialistas em tempo oportuno.

